# Role for Functional *SOD2* Polymorphism in Pulmonary Arterial Hypertension in a Chinese Population

**DOI:** 10.3390/ijerph14030266

**Published:** 2017-03-06

**Authors:** Ming Xu, Min Xu, Lei Han, Chao Yuan, Yong Mei, Hengdong Zhang, Shi Chen, Kai Sun, Baoli Zhu

**Affiliations:** 1 Department of Occupational Disease Prevention, Jiangsu Provincial Center for Disease Control and Prevention, No. 172 Jiangsu Road, Nanjing 210009, China; sosolou@126.com (M.X.); hanlei@jscdc.cn (L.H.); hd-zhang@263.net (H.Z.); 2 Jiangsu Province Official Hospital, Nanjing 210009, China; xumin1212@sina.com; 3 Department of Emergency, the First Affiliated Hospital with Nanjing Medical University, No. 300 Guangzhou Road, Nanjing 210029, China; hayc1987@163.com (C.Y.); njmumy11@163.com (Y.M.); 4 Department of Public Health Sciences, University of North Carolina Charlotte, Charlotte, NC 28223, USA; schen56@uncc.edu

**Keywords:** *SOD2*, exonic polymorphism, susceptibility, pulmonary arterial hypertension

## Abstract

The superoxide dismutase 2 (*SOD2*) gene is a pivotal part of oxidative stress system, which could induce the onset of pulmonary arterial hypertension (PAH). In this study, we quantified the influence of a *SOD2* exonic polymorphism (rs4880) on PAH susceptibility. We genotyped this single nucleotide polymorphism (SNP) by TaqMan, and evaluated its association with PAH susceptibility in a case-control study of 460 patients and 530 controls in China. There were significant differences between PAH cases and controls in both CC and TC+CC genotypes (*p* = 0.013 and *p* = 0.010, respectively). Furthermore, the number of variant alleles followed a dose-response manner (*p* trend was 0.023). Besides, the mRNA level and protein expression also indicated that the C allele of this variant decreased the expression of *SOD2* gene (*p* = 0.004 in mRNA level and *p* = 0.012 in protein level) after the transfection of plasmids containing the different genotype of rs4480. There is significant association between SOD rs4880 polymorphism and the PAH susceptibility, and this polymorphism influenced PAH susceptibility by altering the expression of *SOD2*.

## 1. Introduction

Pulmonary arterial hypertension (PAH) is a severe progressive disease resulting in elevated pulmonary arterial pressure, vascular remodeling, and right ventricular heart failure. In the United States, the number of PAH incident cases is nearly 1000 per year [1]. Although several treatments have been demonstrated to increase survivorship of PAH, a cure for this disease is still far away. Usually, PAH occurs in the 30s and 40s with 66% probability for a 5-year survival [2], ineffective treatment of which is one indication for lung transplants. So far, the mechanisms of PAH have not been comprehensively understood. In 2015 European Respiratory Society (ERS) guidelines, there are several kinds of PAH that are idiopathic, heritable, and drug- and toxin-induced, and are associated with other diseases [3]. According to Stacher’s report, people of all ages could suffer from PAH, including children, and the number of female patients was larger when compared with their male counterparts [4]. Oxidative stress (OS) was considered as an essential factor responsible for PAH [5,6]. In Geraci et al.’s report, abnormal expressions of oxidative genes were found in pulmonary tissues in PAH [7], and the following studies further confirmed the presence of OS in situ in lungs of PAH patients. As the production of OS, the reactive oxygen species (ROS) existed widely in vascular cells, including endothelial cells, smooth muscle cells, and adventitial cells [6]. ROS was closely related to signal transduction and vascular function regulation [8,9]. Dysregulation of ROS may trigger smooth muscle hypercontractility and vascular remodeling during acute lung injury and pulmonary edema [10].

Superoxide dismutase (SOD), an antioxidant enzyme with a high activity on catalytic dismutation of superoxide radical anion, participates in various biological processes induced by OS [11]. Within cells, SOD turns dangerous reactive compounds into oxygen and water, removing stress from oxidation state. It is represented in three forms, named SOD1 (in the plasma), SOD2 (in mitochondria), and SOD3 (in the extracellular matrix). In clinical settings, SOD has been identified to protect cells and extracellular components from damage associated with inflammatory process [12,13], and its protective and beneficial roles have been revealed in numerous diseases [14,15]. Particularly, SOD also reduces the first acute rejection episodes and irreversible acute rejection of cyclosporine-treated recipients of cadaveric renal allograft [16].

Genetic variants of *SOD* genes have been a topic of interest recently. Among three coding genes, single nucleotide polymorphisms (SNPs) of *SOD2* were investigated intensively because of its influence on susceptibility of cancers. rs4880, a well-studied SNP, existed in the exon region of *SOD2* and caused an amino acid substitution. As a consequence, C allele of SNP rs4880 was believed to reduce the SOD2’s transport efficiency in mitochondria; and individuals carrying TT genotype had a higher SOD2 activity level than those who carried TC or CC genotype [17]. Several epidemiological studies investigated the relationship between the SNPrs4880 and lung cancer. However, the influence of this variant on PAH had not been well understood. Considering the consequences of SOD loss on vascular and smooth muscle function, we hypothesized that the functional SNP rs4880 in *SOD2* exon influence the expression of *SOD2*, which was further associated with PAH. To test this hypothesis, we genotyped rs4880 in a case-control study and assessed the genetic association with PAH occurrence by statistical analysis. Furthermore, we validated the regulatory function of rs4880 on *SOD2* by transfection assay.

## 2. Materials and Methods

### 2.1. Ethics Statement

The study protocol was approved by the Institutional Review Board of the First Affiliated Hospital of Nanjing Medical University, Nanjing, China (approval No. 2013-SRFA-007, 26 February 2013). All subjects involved in this study signed the informed consent and agreed to participate in this study voluntarily.

### 2.2. Patients and Controls

In this study, all subjects were in the Han Chinese ethnical group without blood relationship. Patients were enrolled from the First Affiliated Hospital of Nanjing Medical University and Jiangsu Province Official Hospital since January 2006. All patients were primarily diagnosed by pulmonary angiography and right heart catheterization. We used mPAP ≥25 mmHg, pulmonary artery wedge pressure ≤15 mmHg, and elevated pulmonary vascular resistance >3 Wood units as criterion for PAH [18]. Patients with idiopathic, heritable, and associated PAH (groups 1.1, 1.2, and 1.4 of the ERS guidelines from 2015 [3]) were included in the present study. Individuals with unstable atherosclerotic vascular disease, untreated lipidemia, and other chronic lung diseases, which might lead to PAH, were excluded from this study. Healthy subjects, with matched gender and age composition, were enrolled from normal physical examination during the same period, and they formed the control group. Blood samples for DNA extraction were collected immediately after diagnosis without the corresponding drugs were adopt.

### 2.3. DNA Extraction and SNP Genotyping

Genomic DNA was extracted from peripheral blood by DNA blood mini kit (QIAGEN, Hilden, Germany) following manufacturer’s protocol. Genotyping of rs4880 was performed with TaqMan SNP Genotyping Assays (Applied Biosystems, Foster City, CA, USA) by two operators independently and blinded. According to the manufacturer’s instructions, amplifications and analysis were carried out in 384-well ABI 7900HT Real-Time PCR system (Applied Biosystems). Accuracy of genotyping was measured by control samples in each plate. Approximately 10% of samples were randomly repeated for verification and validation. The results were 100% concordant.

### 2.4. Construction of SOD2 Expression Plasmid

In expression plasmid, the human *SOD2* coding sequences (CDS) were correctly synthesized and inserted into pcDNA3.1 vector (Promega, Madison, WI, USA) by Biolight Tec. (Nanjing, China). After further confirmation of DNA sequence and protein product, we performed a single point mutation on rs4880 site to obtain the different plasmid with different alleles.

### 2.5. Cell Culture and Transient Transfection

Human pulmonary artery smooth muscle cell (HPASMc) was purchased from Sciencell Company (San Diego, CA, USA). The smooth muscle cell medium (SMCM) (Gibco/ThermoFisher, Waltham, MI, USA) for HPASMc culturing was at 37 °C in a humidified atmosphere with 95% air and 5% CO_2_.

In transient transfection test, HPASM cells were pre-cultured in Dulbecco’s modified eagle medium (DMEM) (Gibco/ThermoFisher) without serum. Lipofectamine 2000 (Invitrogen, Carlsbad, CA, USA) were adapted to transfect 3.2 μg of each constructed vector into cells in 6-well plate. We changed the complete medium for further culture after six hours.

### 2.6. Expression Levels of SOD2 mRNA and Protein

Total RNA from cells was extracted by Trizol Reagent (Invitrogen, Carlsbad, CA, USA). The mRNA was measured by quantitative real-time PCR (ABI 7500) after reverse transcription, and we chose β-actin as an internal quantitative control for samples. The β-actin as the internal reference, and relative quantification of *SOD2* mRNA was calculated by the 2-ΔΔCt method [(*SOD2* Ct- β-actin Ct in rs4880 C allele) − (*SOD2* Ct- β-actin Ct in rs4880 T allele)].

For Western blot analyses, cells were lysed with Radio Immunoprecipitation Assay (RIPA) lysis buffer. Twenty-five milligrams of total protein were electrophoresed on a 12% SDS-PAGE gel, transferred to a poly vinylidene fluoride (PVDF) membrane (GE Healthcare Life Sciences, Marlborough, MA, USA), and blocked with 10% non-fat milk in Tris-buffered saline and Tween-20 (TBST). The PVDF membrane was incubated with anti-SOD2 (1:1000; Abcam, Cambridge, MA, USA) and anti-β-actin antibody (1:1000; Abcam) overnight at 4 °C to confirm equivalent protein loading in each lane, next with Horseradish Peroxidase (HRP)-conjugated rabbit-anti-mouse IgG for 1 h at room temperature, washed 3 times with TBST for 15 min each, and finally developed with enhanced chemiluminescence (ECL) (Amersham Life Science, Madison, WI, USA) on X-ray film.

### 2.7. Statistical Analysis

Data analyses were carried in SAS (version 9.2, SAS Institute Inc., Cary, NC, USA). Hardy–Weinberg equilibrium (HWE) was evaluated by a goodness-of-fit chi-square to ensure the effectiveness of SNP allele frequency. Logistic regression analyses, adjusted for age and sex, were performed to estimate the association between SNP rs4880 and risk of PAH. A Student’s *t*-test was used to evaluate the expression of *SOD2* in peripheral blood, and the 6-min walk distance (6MWD), N-terminal pro B-type natriuretic peptide (NT-proBNP), mean pulmonary artery pressure (mPAP), cardiac output (CO), and cardiac index (CI) in PAH patients. The non-parametric test was used for the pulmonary vascular resistance index (PVRI) in the stratification analysis of PAH patients because of its abnormal distribution. The trend of influence by variants in alleles was tested as a*p* trend. Two-tailed (α = 0.05) and *p* < 0.05 was adopted as statistically significance in this study.

## 3. Results

### 3.1. Characteristics of Study Subjects

The demographic information of 460 patients (cases) and 530 healthy controls are summarized in Table 1. Both age and gender were well-matched between cases and controls without significant differences (*p* = 0.915 and 0.853, respectively). There was no significant difference between cases and controls in Body Mass Index (BMI) (*p* = 0.399) either. The median of 6 min walking distance (6MWD) was 247.9 m for patients. The NT-proBNP, mPAP, and PVRI are also provided in Table 1 with median values (1139.9 pg/mL, 51 mmHg, and 1485.2 dyn*sec*m^2^/cm^5^, respectively).

### 3.2. Genotype Frequencies of SOD2 Polymorphism in PAH Cases and Controls

The genotypes of *SOD2* rs4880 are shown in Table 2. The frequencies of CC and CC + TC in patients were significantly different from controls with *p* = 0.013 and *p* = 0.010, respectively. Compared with TT individuals (as reference), individuals with TC + CC genotypes had a substantial increased susceptibility for PAH incidence (OR = 2.75, 95% CI = 1.23–3.98, *p* = 0.010). These results suggested that *SOD2* rs4880 had a strong influence on PAH occurrence.

### 3.3. Correlation with Clinical Features and SOD2 rs4880

The associations between clinical characters and *SOD* rs4880 in PAH patients were shown in Table 3. With polymorphism in dominant model, the TC + CC displayed the statistically decreased trends in 6MWD (TT vs. TC + CC = 258.7 ± 49.6 m vs. 239.2 ± 41.3 m, *p* = 0.001), CO (TT vs. TC + CC = 4.01 ± 1.84 L/min vs. 3.58 ± 1.73 L/min, *p* = 0.021), and CI (2.36 ± 0.79 L/min/m^2^ vs. 2.18 ± 0.91 L/min/m^2^, *p* = 0.035). On the opposite, the NT-proBNP and mPAP demonstrated an increased trend in TC + CC group (both *p* < 0.001). In addition, there is no significance in the PVRIs between TT groups and TC + CC groups in PAH patients.

### 3.4. Effect of SOD2 Polymorphism on SOD2 Transcription

**Figure 1:**
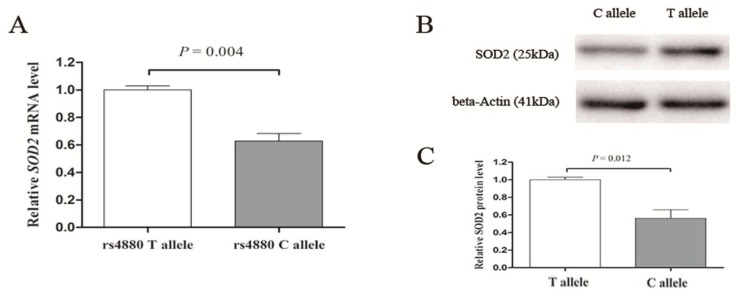
*SOD2* expression levels of rs4880 different variants. (**A**) *SOD2* mRNA expression; (**B**) SOD2 protein expression; (**C**) Gray scale analysis of (B).

To investigate whether rs4880 polymorphism influence *SOD2* transcription, we transfected two different expression plasmids (T allele as wild type and C allele as mutate type) into HPASMc. After both mRNA and protein expression were detected, we found that the C allele was associated with reduced *SOD2* mRNA level and decreasing SOD2 protein as well (Figure 1).

## 4. Discussion

In this study, we identified and quantified the significant association between *SOD2* SNP rs4880 in exon region and PAH susceptibility. Our study suggested that C allele (or Ala) were significantly associated with PAH susceptibility. Besides, we also found that C allele was closely related to the lower expression of *SOD2*. To the best of our knowledge, this is the first report that investigated the influence of *SOD2* polymorphism on PAH susceptibility.

SOD2, the major form of SODs expressed in mitochondria, participated extensively in cellular processes, such as metabolism [19,20], progression [21], proliferation [22,23], invasion [24], and apoptosis [25,26]. In PAH, the dysfunctional angiogenesis was a critical biologic event leading to lung vessel obliteration [27]. In Bowers’ report, the lung tissue from severe pulmonary hypertension (PH) patients undergoes oxidant stress, accompanying decreased amounts of Mn-SOD protein and decreased Mn-SOD activity [28]. These variations could induce both overproduction of superoxide anion and impaired enzymatic removal of the oxidant in PH lungs [28].

There are multiple regulatory mechanisms of *SOD2*: both genetics and epigenetics alterations cooperate in its dysregulation in cells. In a recent study [29], *SOD2* possessed three distinct kinds of elements-CpG island, intronic enhancer, and numerous regulatory elements, all of which ensured the dynamic regulation of *SOD2* undertaking changes in cellular metabolism, exogenous stimuli, and abiotic stresses. In the same article, the changes of DNA methylation and histone modification were also identified in carcinogenesis [29]. Because of the interference elimination of these factors, our findings provide evidence that *SOD2* rs4880 T/C alleles not only induced the exchange from Val to Ala, but also decreased the expression of *SOD2* in HPASMc. Comparing with T plasmid, the *SOD2* mRNA level substantially decreased when cells were transfected with C plasmid. It suggested that a variation in exon could also trigger certain mechanisms and regulated gene expression during the transcriptional process in PAH, which might be a novel regulation method in *SOD2* in PAH.

Until now, no similar research has been performed on PAH. However, for other lung diseases, such as lung cancer, the influence of rs4880 was controversial. Four studies described that wild-type TT genotype of *SOD2* rs4880 T/C polymorphism was associated with an increased lung cancer risk in Caucasian population, while two on Asian population did not identify similar association even after stratification analysis. Besides, a large-scale study confirmed the impact of rs4880 C variant with a *p*-trend of 0.04. However, the lung carcinoma was a more complicated lung disease, including invasion and metastasis, which did not exist in PAH. Clinically, PAH was considered as vascular dysplasia [30]. Meanwhile, the influence of SOD2 induced by vasohibin-1 (an endothelial cell-produced angiogenesis inhibitor) was identified to prevent acute lung injury [31]. Our results also revealed that the C allele of *SOD2* rs4880 was associated with an increased incidence of PAH among Chinese population, and this SNP could be a susceptibility biomarker for PAH.

Nevertheless, some potential limitations of this study existed. First, only 430 patients of PAH were enrolled. The relatively small sample size might cause lower statistical power in our study. Besides, we used plasmid to reduce the influences of other regulation methods in PAH; however, these influences could not be completely eliminated. To some extent, these factors also regulated the expression of *SOD2*. A comprehensive investigation of both genetics and epigenetic factors will be the future direction for the systematic analysis on *SOD2* expression regulation. In addition, rs4880 was not the only polymorphism in *SOD2*, and the comprehensive analysis among SNPs needs to be carried out in successive studies to study their potential interactions.

## 5. Conclusions

We identified significant differences of *SOD2* rs4880 polymorphism between PAH patients and controls. *SOD2* rs4880 polymorphism could influence PAH susceptibility by reducing SOD protein expression.

## Figures and Tables

**Table 1:** Demographic and hemodynamic characteristics of PAH patients (case) and normal subjects (control).

Variables	Case (*n* = 460)	Control (*n* = 530)	*p*
age (years)	44.6 ± 13.9	45.4 ± 13.6	0.861
sex			0.853
male	135 (29.3%)	163 (30.8%)	
female	325 (70.7%)	367 (69.2%)	
BMI (kg/m^2^)	25.6 ± 4.5	25.1 ± 5.3	0.399
6MWD (m)	247.9 (146.2–363.3)		
NT-proBNP (pg/mL)	1139.9 (360.9–2471.8)		
mPAP (mmHg)	51 (40–59)		
PVRI (dyn*sec*m^2^/cm^5^)	1485.2 (1137.3–2742.7)		
CO (L/min)	3.86 ± 1.81		
CI (L/min/m^2^)	2.23 ± 0.88		

Data are presented as mean ± SD or median (interquartile range). BMI: body mass index; 6MWD: 6-min walk distance; NT-proBNP: N-terminal pro brain natriuretic peptide; mPAP: mean pulmonary arterial pressure; PVRI: pulmonary vascular resistance index; CO: cardiac output; CI: cardiac index.

**Table 2:** Genotype frequencies of *SOD2* rs4880 polymorphism in PAH cases and controls.

rs4880 T > C	Cases, *n* (%)	Controls, *n* (%)	OR (95% CI) ^a^	*p* ^a^
TT	319 (69.35%)	426 (80.37%)	Reference	-
TC	111 (24.13%)	99 (18.68%)	1.35 (0.79–2.81)	0.195
CC	30 (6.52%)	5 (0.94%)	3.81 (1.22–5.67)	0.013
TC + CC	141 (30.65%)	104 (19.62%)	2.75 (1.23–3.98)	0.010
*p* trend				0.023

^a^ adjusted for age and sex in additive model in logistic regression.

**Table 3:** Stratified analyses of *SOD2* rs4880 polymorphism associated with the PAH clinical features by genetic dominant model.

Variables	Case (*n* = 460)		*p* Value ^a^
	TT (*n* = 319)	TC + CC (*n* = 141)	
6MWD (m)	258.7 ± 49.6	239.2 ± 41.3	0.001
NT-proBNP (pg/mL)	1075.7 ± 256.5	1177.8 ± 243.4	<0.001
mPAP (mmHg)	50 ± 5	52 ± 7	<0.001
PVRI (dyn*sec*m^2^/cm^5^)	1461.6 ± 252.9	1516.0 ± 274.2	0.063 ^b^
CO (L/min)	4.01 ± 1.84	3.58 ± 1.73	0.021
CI (L/min/m^2^)	2.36 ± 0.79	2.18 ± 0.91	0.035

^a^ Adjusted for age, sex, and BMI in logistic regression model. ^b^ Non-parametric test were performed for PVRI.

## References

[1] U.S. National Library of Medicine Genetics Home. http://ghr.nlm.nih.gov/condition/pulmonary-arterial-hypertension.

[2] Thenappan T., Shah S.J., Rich S., Tian L., Archer S.L., Gomberg-Maitland M. (2010). Survival in pulmonary arterial hypertension: A reappraisal of the NIH risk stratification equation. Eur. Respir. J..

[3] Galie N., Humbert M., Vachiery J.L., Gibbs S., Lang I., Torbicki A., Simonneau G., Peacock A., Vonk Noordegraaf A., Beghetti M. (2016). 2015 ESC/ERS Guidelines for the Diagnosis and Treatment of Pulmonary Hypertension. Eur. Heart J..

[4] Stacher E., Graham B.B., Hunt J.M., Gandjeva A., Groshong S.D., McLaughlin V.V., Jessup M., Grizzle W.E., Aldred M.A., Cool C.D. (2012). Modern age pathology of pulmonary arterial hypertension. Am. J. Respir. Crit. Care Med..

[5] Haase E., Bigam D.L., Nakonechny Q.B., Rayner D., Korbutt G., Cheung P.Y. (2005). Cardiac function, myocardial glutathione, and matrix metalloproteinase-2 levels in hypoxic newborn pigs reoxygenated by 21%, 50%, or 100% oxygen. Shock.

[6] Zhang S., Yang T., Xu X., Wang M., Zhong L., Yang Y., Zhai Z., Xiao F., Wang C. (2015). Oxidative stress and nitric oxide signaling related biomarkers in patients with pulmonary hypertension: A case control study. BMC Pulm. Med..

[7] Geraci M.W., Moore M., Gesell T., Yeager M.E., Alger L., Golpon H., Gao B., Loyd J.E., Tuder R.M., Voelkel N.F. (2001). Gene expression patterns in the lungs of patients with primary pulmonary hypertension: A gene microarray analysis. Circ. Res..

[8] Heiss E.H., Schachner D., Werner E.R., Dirsch V.M. (2009). Active NF-E2-related factor (Nrf2) contributes to keep endothelial NO synthase (eNOS) in the coupled state: Role of reactive oxygen species (ROS), eNOS, and heme oxygenase (HO-1) levels. J. Biol. Chem..

[9] Roth T.L., Nayak D., Atanasijevic T., Koretsky A.P., Latour L.L., McGavern D.B. (2014). Transcranial amelioration of inflammation and cell death after brain injury. Nature.

[10] Birukov K.G. (2009). Cyclic stretch, reactive oxygen species, and vascular remodeling. Antioxid. Redox Signal..

[11] McCord J.M., Fridovich I. (1969). Superoxide dismutase. An enzymic function for erythrocuprein (hemocuprein). J. Biol. Chem..

[12] Salin M.L., McCord J.M. (1975). Free radicals and inflammation. Protection of phagocytosine leukocytes by superoxide dismutase. J. Clin. Investig..

[13] Shuvaev V.V., Han J., Yu K.J., Huang S., Hawkins B.J., Madesh M., Nakada M., Muzykantov V.R. (2011). PECAM-targeted delivery of SOD inhibits endothelial inflammatory response. FASEB J..

[14] Sharpe M.A., Ollosson R., Stewart V.C., Clark J.B. (2002). Oxidation of nitric oxide by oxo manganese-salen complexes: A new mechanism for cellular protection by superoxide dismutase/catalase mimetics. Biochem. J..

[15] Robbins D., Zhao Y. (2014). Manganese superoxide dismutase in cancer prevention. Antioxid. Redox Signal..

[16] Land W., Schneeberger H., Schleibner S., Illner W.D., Abendroth D., Rutili G., Arfors K.E., Messmer K. (1994). The beneficial effect of human recombinant superoxide dismutase on acute and chronic rejection events in recipients of cadaveric renal transplants. Transplantation.

[17] Sutton A., Khoury H., Prip-Buus C., Cepanec C., Pessayre D., Degoul F. (2003). The Ala16Val genetic dimorphism modulates the import of human manganese superoxide dismutase into rat liver mitochondria. Pharmacogenetics.

[18] Galie N., Hoeper M.M., Humbert M., Torbicki A., Vachiery J.L., Barbera J.A., Beghetti M., Corris P., Gaine S., Gibbs J.S. (2009). Guidelines for the diagnosis and treatment of pulmonary hypertension. Eur. Respir. J..

[19] Costa F., Dornelles E., Manica-Cattani M.F., Algarve T.D., Filho O.C.S., Sagrillo M.R., Garcia L.F., Cruz I.B. (2012). Influence of Val16Ala *SOD2* polymorphism on the in vitro effect of clomiphene citrate in oxidative metabolism. Reprod. Biomed. Online.

[20] Martin F.M., Xu X., von Lohneysen K., Gilmartin T.J., Friedman J.S. (2011). SOD2 deficient erythroid cells up-regulate transferrin receptor and down-regulate mitochondrial biogenesis and metabolism. PLoS ONE.

[21] Miar A., Hevia D., Munoz-Cimadevilla H., Astudillo A., Velasco J., Sainz R.M., Mayo J.C. (2015). Manganese superoxide dismutase (SOD2/MnSOD)/catalase and SOD2/GPx1 ratios as biomarkers for tumor progression and metastasis in prostate, colon, and lung cancer. Free Radic. Biol. Med..

[22] Wei L., Zhou Y., Qiao C., Ni T., Li Z., You Q., Guo Q., Lu N. (2015). Oroxylin A inhibits glycolysis-dependent proliferation of human breast cancer via promoting SIRT3-mediated *SOD2* transcription and *HIF1alpha* destabilization. Cell Death Dis..

[23] Liu J., Narasimhan P., Lee Y.S., Song Y.S., Endo H., Yu F., Chan P.H. (2006). Mild hypoxia promotes survival and proliferation of SOD2-deficient astrocytes via c-Myc activation. J. Neurosci..

[24] Liu Z., He Q., Ding X., Zhao T., Zhao L., Wang A. (2015). *SOD2* is a C-myc target gene that promotes the migration and invasion of tongue squamous cell carcinoma involving cancer stem-like cells. Int. J. Biochem. Cell Biol..

[25] Drane P., Bravard A., Bouvard V., May E. (2001). Reciprocal down-regulation of p53 and *SOD2* gene expression-implication in p53 mediated apoptosis. Oncogene.

[26] Kokoszka J.E., Coskun P., Esposito L.A., Wallace D.C. (2001). Increased mitochondrial oxidative stress in the SOD2 (+/−) mouse results in the age-related decline of mitochondrial function culminating in increased apoptosis. Proc. Natl. Acad. Sci. USA.

[27] Voelkel N.F., Gomez-Arroyo J. (2014). The role of vascular endothelial growth factor in pulmonary arterial hypertension. The angiogenesis paradox. Am. J. Respir. Cell Mol. Biol..

[28] Bowers R., Cool C., Murphy R.C., Tuder R.M., Hopken M.W., Flores S.C., Voelkel N.F. (2004). Oxidative stress in severe pulmonary hypertension. Am. J. Respir. Crit. Care Med..

[29] Cyr A.R., Hitchler M.J., Domann F.E. (2013). Regulation of *SOD2* in cancer by histone modifications and CpG methylation: Closing the loop between redox biology and epigenetics. Antioxid. Redox Signal..

[30] Miyashita H., Watanabe T., Hayashi H., Suzuki Y., Nakamura T., Ito S., Ono M., Hoshikawa Y., Okada Y., Kondo T. (2012). Angiogenesis inhibitor vasohibin-1 enhances stress resistance of endothelial cells via induction of *SOD2* and *SIRT1*. PLoS ONE.

[31] Orlova V.V., Liu Z., Goumans M.J., ten Dijke P. (2011). Controlling angiogenesis by two unique TGF-beta type I receptors signaling pathways. Histol. Histopathol..

